# Cognitive alterations related to driving performance in Parkinson’s disease detected by a driving simulator

**DOI:** 10.1038/s41598-025-31585-y

**Published:** 2025-12-09

**Authors:** Almudena Cerezo-Zarzuelo, Francisco José Sánchez-Cuesta, Carlota Trigo, Eduardo Rocon, Jorge Villagra, Juan Felipe Medina-Lee, Vinicius Trentin, Juan Pablo Romero

**Affiliations:** 1 Escuela Internacional de Doctorado (EIDUNED), Universidad Nacional de Educación a Distancia (UNED), Bravo Murillo 38 St, 28015 Madrid, Spain; 2 Brain Injury and Movement Disorders Neurorehabilitation Group (GINDAT), Institute of Life Sciences, Universidad Francisco de Vitoria, Ctra. Pozuelo-Majadahonda Km. 1.800, 28223 Pozuelo de Alarcón, Madrid Spain; 3 Brain Damage Unit, Beata María Ana Hospital, Dr. Esquerdo 83 St, 28007 Madrid, Spain; 4 Faculty of Experimental Sciences, Universidad Francisco de Vitoria, 28223 Pozuelo de Alarcón, Madrid Spain; 5 Centre for Automation and Robotics (CSIC-UPM), Ctra. de Campo Real km. 0.200, 28500 Arganda del Rey, Spain; 6 Computer Science and Engineering Department, University of Puerto Rico, 259 Av. Alfonso Valdés Cobián, 00680 Mayagüez, Puerto Rico

**Keywords:** Parkinson disease, Driving simulation, Neuropsychological tests, Reaction time, Diseases, Neurology, Neuroscience, Psychology, Psychology

## Abstract

Driving ability in individuals with Parkinson’s disease (PD) can be compromised early in the course of the illness, even before clear cognitive deficits emerge on standard neuropsychological tests. This study investigated subtle driving impairments in a group of non-demented people with PD using a high-fidelity driving simulator. Seven PD participants and seven healthy controls, matched for age and sex, completed cognitive assessments, reaction time tasks, and five simulated driving scenarios that measured lane keeping, steering control, and reaction to events. While most cognitive scores were comparable between groups, PD participants exhibited slower response times in basic tasks and showed reduced lane control, particularly during left turns. These difficulties were associated with disease severity and medication dosage. The simulator proved more sensitive than conventional tests in detecting early impairments related to attention and visuospatial processing. These findings suggest that driving simulators may play a key role in improving the assessment of driving competence in PD, providing insight into real-world challenges faced by this population.

## Introduction

Driving is a complex task that requires the integration of multiple perceptual, motor, and cognitive processes, with cognitive function being essential for effective real-time decision-making, safe responses and behavioural adaptation to dynamic environments^1^. From a psychological perspective, safe driving depends on multiple higher-order processes such as divided attention, executive control, visuospatial processing, and risk assessment. These abilities enable drivers to maintain situational awareness, predict the behaviour of other road users, and regulate their own responses under varying levels of cognitive and emotional load^2,3^. In most countries, fitness to drive is evaluated through standardized psychotechnical assessments that include a variety of tasks aimed at measuring visual perception, reaction time, coordination, and attention. Many of these variables are directly influenced by cognitive functioning.

In Parkinson’s disease (PD), a neurodegenerative disorder marked by dopaminergic circuit degeneration, motor symptoms such as rigidity, rest tremor, and bradykinesia are usually the basis of the diagnosis, yet cognitive dysfunctions—including deficits in attention, executive function, and working memory—are often present since early stages^4^. These impairments not only affect cognitive performance but also influence driving behavior, reducing the ability to manage multitasking, inhibit impulsive actions, and adapt to unexpected traffic event^2,3,5^. As a result, individuals with PD may struggle to respond efficiently to dynamic traffic situations, such as reacting to signals, avoiding obstacles, or coordinating interactions with other vehicles^5^. They frequently demonstrate delayed reaction times and reduced accuracy in tasks requiring quick decisions and multitasking, which increases the risk of collisions, particularly in complex driving environments^1,6,7^. Moreover, diminished executive function further impairs planning and adaptability to unexpected events, thereby undermining overall fitness to drive^8^.

Standard driving assessment exams generally include tests for reaction time, visual processing, attention, and motor coordination, domains frequently impaired in PD patients. In Spain, standard psychotechnical driving assessments include basic medical examinations, like checks of visual and auditory acuity and the management of chronic conditions, including neurological disorders. However, these rarely involve specific cognitive evaluations or test modifications. These screenings are complemented by automated testing in a psychotechnical cabin, usually involving a screen and manual controls. The most common tasks assess visuomotor coordination (e.g., maintaining a ball centered within a moving lane using two joysticks) and simple reaction time to visual or auditory stimuli. While these tests aim to evaluate essential driving-related skills such as coordination, reaction time, and anticipatory capacity, they are performed in abstract, decontextualized environments that fail to replicate the complexity and realism of actual driving situations.

A recent systematic review by Stamatelos et al.^9^ emphasized that while international guidelines recognize the impact of cognitive decline in PD on driving performance, there is considerable variability in how fitness to drive is assessed across countries. The review also highlighted a lack of consensus regarding the most reliable cognitive tools for driving evaluation and called for more evidence-based, individualized approaches that account for the heterogeneous progression of PD and its cognitive symptoms.

Assessing fitness to drive in individuals with PD requires specific tools that evaluate the cognitive and psychomotor functions crucial to driving safety. In an era of increasing automation in driving and the development of new technologies that enable more immersive experiences and control over a wider range of variables, it is possible to identify safety indicators behind the wheel that may be overlooked in standard neuropsychological assessments or superficial evaluations of reaction times. As a response to these challenges, driving simulators have emerged as a promising alternative, allowing for detailed and controlled measurement of key driving parameters, such as trajectory accuracy and lane-centered positioning^10–12^.

This study aims to identify objective differences in key driving safety variables between individuals with Parkinson’s disease (PD) and healthy controls using a driving simulator. We hypothesize that PD patients will show altered reaction times and reduced vehicle control precision compared to controls. By providing empirical evidence, this study seeks to support future efforts toward the development of more objective tools for assessing driving fitness, taking into account the specific neuropsychological profiles associated with different neurodegenerative diseases.

## Methods

### Participants

Seven drivers with PD were recruited and matched in age and gender with seven healthy regular drivers. There were no significant differences between groups regarding years of driving experience (Patients: 43.57 years ± 8.84; Controls: 44.86 years ± 8.70), daily driving distance average (Patients: 20.42 km ± 15.84; Controls: 42.86 km ± 21.57) nor frequency of driving (Patients: 3.43 days ± 2.23, Controls: 5 days ± 1.77) per week. Selection criteria for participants with PD were: (1) PD diagnosis, (2) younger than 75 years old, (3) they must hold a driver’s license and have driving experience, (4) no changes in medication for the past 30 days and (5) attend the evaluations on ON state (1 h after last oral dose of levodopa), (6) a Hoehn&Yahr stage lower or equal to 3, (7) a punctuation higher or equal to 24 in the Montreal Cognitive Assessment (MoCA), (8) not having visuoperceptual impairments. The Unified Parkinson’s Disease Rating Scale (UPDRS) was assessed for each patient, to assure the ON state when conducting the evaluation.

The selection criteria for the control participants were: (1) holding a driver’s license and driving experience, (2) younger than 75 years and (3) not having visuoperceptual impairments.

The recruitment, as well as the evaluation, were conducted in the Beata Maria Ana Hospital, from March 1st to March 12th and June 3rd to June 14th, 2024. All participants were informed of the details of the evaluation and signed their consent to participate in this study, in accordance with the declaration of Helsinki. The Ethics Committee of the 12 de Octubre Hospital approved the study on 6th February 2024 with code 23/603.

### Experimental design and procedures

This is a proof-of-concept study with a cross-sectional measurement in which our main objective was to assess methodological feasibility and reliability of using a driving simulator to detect differences in PD patients that still drive. Evaluations of all the participants were carried out in a single session with two different parts: a cognitive assessment and the driving simulation tests, with a total duration of 120 min. Demographic and clinical data from participants are shown in Tables 1 and 2.

**Table 1:** Participants’ demographic characteristics.

Participant	Gender	Age (years)	Laterality
Control 1	Masculine	60	Right-handed
Control 2	Masculine	74	Right-handed
Control 3	Masculine	60	Right-handed
Control 4	Masculine	63	Right-handed
Control 5	Masculine	66	Right-handed
Control 6	Masculine	59	Right-handed
Control 7	Masculine	74	Right-handed
Experimental 1	Masculine	71	Right-handed
Experimental 2	Masculine	69	Right-handed
Experimental 3	Masculine	66	Right-handed
Experimental 4	Masculine	56	Right-handed
Experimental 5	Masculine	70	Right-handed
Experimental 6	Masculine	54	Right-handed
Experimental 7	Masculine	63	Right-handed

**Table 2:** Clinical characteristics of the participants with PD.

Participant	Years since diagnosis	Hoehn and Yahr stage	Levodopa equivalents daily dose (mg)
Experimental 1	1	2	400
Experimental 2	5	2	1125
Experimental 3	7	2	1050
Experimental 4	3	1.5	300
Experimental 5	11	2.5	1050
Experimental 6	3	2	791.08
Experimental 7	9	2	2074.8

#### Cognitive assessment

All participants completed a battery of the following neuropsychological tests prior to the driving simulation tests for 60 min:

- MoCA: it is a brief screening tool for general cognition functioning^13^.
- Stroop test: this test measures cognitive flexibility, selective attention, cognitive inhibition and information processing speed. It includes four different parts: words, colour, words-colour and interference^14^.
- Wechler adult intelligence scale (WAIS-IV): This battery is the gold standard for the evaluation of cognitive abilities in adults. We selected two tests from this battery: symbol search and digit-symbol substitution to assess information processing speed, visual attention, work memory and incidental learning^15^.
- Computerized reaction time tasks: reaction time tasks have been previously used with patients with PD when assessing deficits in the information processing^16^. We performed two tasks, using a 27-inch monitor, controlled by Presentation^®^ software (Neurobehavioral Systems Inc, Albany, California, United States)^17^, based on the Arroyo et al. study^16^. The average reaction time in each of the tasks and the percentage of correct answers were measured. The two tasks were displayed in the following order:
  - Finger tapping (FT): The FT task is a common test of sensory-motor performance^18^. It has been applied following the Strauss application norms: participants were asked to press the keyboard spacebar as repeatedly with the index finger as fast as they can, with 5 attempts with each hand in 10-seconds trials. The collected data is the average time between taps.
  - Simple reaction time (SRT): It is a test designed to measure simple perception and sustained alertness. Participants were asked to press the left mouse button as fast as they can when a “+” appeared in the centre of the screen. It involved 50 trials with a total duration of 2–3 min.
  - Simple reaction time-sustained attention to response task (SRT-SART): This task was applied to measure response strategy-inhibition. It consists of 168 Go trials and 21 No/Go trials, with a duration of 4 min. The stimulus size varied between 12 and 9 mm.
  - Choice reaction time (CRT): This task was employed to measure visual perceptual decision time. It adds the processing of uncertainty to the processes involved in the SRT. Participants were asked to press the left button of the mouse when they see a square at the centre of the screen, and the right button when a circle appeared. It included 80 tests with a duration of 3 min.
  - Choice reaction time-search (CRT-Search): This task was applied to measure visual search. Participants were asked to press the left button of the mouse when they see a “Z” in a 6-letter sequence, or to press the right one if there was no “Z”.

Stimuli were classified in two groups: whether there was a “Z” or not and the visual characteristics of the other letters of the sequence. Therefore, four different combinations were obtained: target-low interference, target-high interference, non-target-low interference and no-target-high interference. The task involved 128 trials, executed between 5 and 8 min.

#### Driving simulation

The driving simulator was displayed on a computer with three 27-inch screens. In this setup, the driver sits approximately 0.9 m from the central screen, allowing a field of view of around 130º, which enables safe management of scenarios such as intersections and roundabouts. Interaction with the simulation software is achieved using the Logitech G29 kit (Logitech, Laussane, Switzerland), which includes a force-feedback steering wheel and a pedal set with realistic resistance. The chair can be configured to adjust both height and distance, ensuring each driver feels comfortable reaching the pedals and steering wheel.

**Fig. 1:**
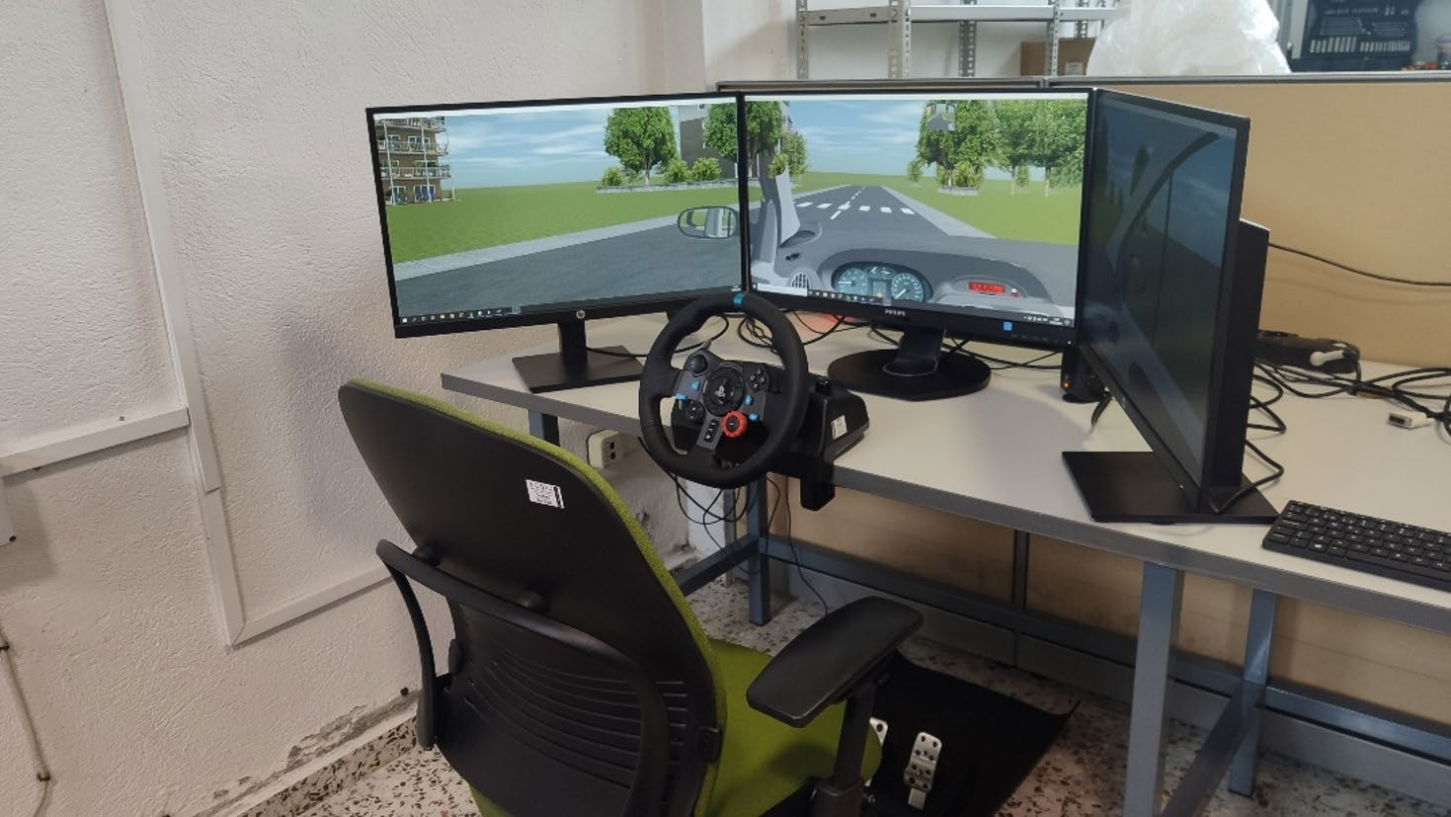
Driving simulator setup used in the experiments, consisting of a steering wheel with pedals, three monitors displaying the virtual driving environment, and a participant’s chair. Driving simulator setup used in the study. The system includes a steering wheel with pedals, three monitors providing a wide field of view of the virtual driving environment, and a participant’s chair positioned in front of the workstation. This setup was used to conduct all trials and to record participants’ driving performance under controlled conditions.

The simulation software used was the SCANeR Studio by AV Simulation (AVSimulation, Boulogne-Billancourt, France)^19^. This automotive simulation software, commercially available, allows for the assessment of driving performance by humans and autonomous driving systems. The tool provides all the necessary modules to construct a realistic virtual environment, including road settings, vehicle dynamics, traffic and sensor configurations, weather conditions, and scenario customization (Fig. 1). This software is executed on a computer with an Intel Core i7-7700 3.6 GHz processor and 16 GB RAM.

The simulation protocol includes five experiments assessing different aspects related to fitness to drive, which are executed in 45–60 min. From the driving experiments, a set of metrics was obtained to measure driving performance objectively. These metrics, which include reaction times, lane deviation, distance to the leader vehicle, and relative speeds, are widely used in the literature to evaluate the performance of drivers in simulation-based studies^28–31^.

Experiment 1:

Participants were asked to follow the car in front of them at a constant speed and to stop the movement when the other car stops. The task consists of initiating movement and maintaining a constant speed following a straight line. At the end of the experiment, the lead car stops at an aleatory time to test the participant’s reaction.

- Metrics derived: Reaction time, defined as the time difference between when the simulated car begins to brake and when the participant initiates braking (s).

Experiment 2:

Participants were asked to follow the car in front of them and adapt their speed to match the pace of the lead vehicle. At the end of the experiment, the car in front stops at an aleatory time to test the participant’s reaction.

- Metrics derived: (1) Reaction time, defined as the time difference between when the simulated car begins to accelerate and when the participant begins to accelerate (s) measured only at the beginning of the experiment. (2) Mean distance between the simulated car and the participant’s car (m). (3) Mean difference in velocity between the simulated car and the participant’s car (km/h).

Experiment 3:

Participants were asked to make a right turn from the right lane without oncoming traffic. They must initiate the movement, approach the intersection, execute a tight right turn without trespassing the left lane, and stop at a specific spot.

- Metrics derived: (1) Percentage of time the car remained within the lane (%). (2) Maximum lateral distance from the centre of the lane (m). (3) Area between the edge of the road and the vehicle’s trajectory outside the lane (m^2^).

Experiment 4:

Participants were asked to make a left turn from the left lane without oncoming traffic. They must initiate the movement, approach the intersection, execute a wider left turn while maintaining the car within the left lane, and stop at a specific spot.

- Metrics derived: (1) Percentage of time the car remained within the lane (%). (2) Maximum lateral distance from the centre of the lane (m). (3) Area between the edge of the road and the vehicle’s trajectory outside the lane (m^2^).

Experiment 5:

Participants were asked to make a reverse turn covering approximately 10 m and stop in a specific spot without oncoming traffic.

- Metrics derived: (1) Percentage of time the car remained within the lane (%). (2) Maximum lateral distance from the centre of the lane (m). (3) Area between the edge of the road and the vehicle’s trajectory outside the lane (m^2^).

Experiments 1 and 2 aim to assess the driver’s ability to adapt dynamically to a controlled traffic environment, paying attention to the stimulus from the vehicle before them. Experiment 1 specifically assesses selective and sustained attention, processing speed, inhibitory control, and executive functions. Experiment 2 examines divided attention, working memory, motor planning, and cognitive flexibility related to motor initiation.

Experiments 3 and 4 assess the driver’s ability to perform turns at intersections—to the right and left, respectively—while maintaining a centred position within the lane during the turn.

Experiment 5 assesses the ability to make a turn while driving in reverse. These tasks require precise integration of visuospatial planning, visuomotor coordination, attentional control, and executive functions.

All experiments are designed to detect cognitive impairments commonly observed in Parkinson’s disease, which can compromise motor adaptation and driving safety. The interpretation of the experiments is carried out considering Spanish drivers drive on the right side of the road.

### Statistical analysis

Differences between control participants and PD patients were determined through Student’s t and Wilcoxon tests regarding demographic variables, driving experience, driving distance average and frequency of driving per week, neuropsychological tests, reaction time tasks and variables involved in the driving simulator. For multiple comparisons, the level of significance was adopted *p* < .05.

An ANCOVA was used to identify the cognitive components contributing to the SRT Task: the slowness in the processing of the information associated with the perceptual and sustained alert components was analysed with the SRT task as the dependent variable and the response time in the FT task as the covariate. Use of FT as a covariate allows controlling the shared “motor” component with the STR task. The significance level was adopted *p* < .05.

To compare the experiment execution in experimental and control group, linear mixed models were applied. This approach was selected to account for the repeated measures structure of the data, as multiple trials were collected per participant. In the models, group (control, experimental) was included as a fixed effect while subject was modelled as a random effect to account for within-subject variability.

Spearman correlations were performed with the PD patients’ data between variables considered in the driving simulation and the different reaction time tasks, and PD-related variables: the HY stage, years of evolution of the disease and levodopa equivalents daily dose. The significance level was adopted *p* < .05. Analyses were performed using R (4.4.2. version, Posit, Boston, MA, United States) and MATLAB (2023b version, MathWorks, Natick, MA, United States)^20^.

## Results

### Cognitive assessment

#### Neuropsychological tests

Wilcoxon tests showed no significant differences between groups in the MoCA (W = 15 [− 3.99 − 1.99]; *p* = 0.14) nor Stroop test (words: W = 21.5 [− 38.00–22.00]; *p* = 0.49; colour: W = 29.5 [− 8.99–10.99], *p* = 0.90; words-colour: W = 19.5 [− 21.99–8.99]; *p* = 0.35; interference: W = 17 [− 16.30–1.45]; *p* = 0.22) between control and experimental groups. Student´s t tests were used with scalar punctuations of symbol search (t = 2.5955 [0.37–4.21]; *p* = 0.02354) and digit-symbol (t = 1.1793 [− 1.38–4.52]; *p* = 0.2645), which showed a significant difference between groups in symbol search punctuations.

**Table 3:** Neuropsychological tests results.

Neuropsychological test	Control group	Experimental group	Statistic	*P* value
MoCA	27.72	26.25	W = 15 (− 3.99−1.99)	0.14
Stroop test				
Words	115.43	108.13	W = 21.5 (− 38.00−22.00)	0.49
Colour	73.71	74.13	W = 29.5 (− 8.99−10.99)	0.90
Words-colours	52.57	45.25	W = 19.5 (− 21.99−8.99)	0.35
Interference	6.96	0.55	W = 17 (− 16.30–1.45)	0.22
Symbol search*	29.71/13.43	24.5/11.14	t = 2.6 (0.37–4.21)	0.02*
Digit-symbol substitution	57.29/12.43	50.75/10.86	t = 1.2 (− 1.38–4.52)	0.26

#### Reaction time tasks

Wilcoxon tests revealed no significant differences between groups in FTT (non-dominant hand: W = 32 [− 27.89–45.80]; *p* = 0.69; dominant hand: W = 26 [− 23.63–23.58]; *p* = 0.87), SRT-SART (average time: W = 36 [− 26.85–79.41]; *p* = 0.40; correct answers: W = 22.5 [− 3.99–1.99]; *p* = 0.55), CRT (average time: W = 36 [− 26.85–79.41]; *p* = 0.40; correct answers: W = 22.5 [− 3.99–1.99]; *p* = 0.55) and CRT-Search (average time: W = 28 [− 93.18–119.61]; *p* = 1; correct answers: W = 12.5 [− 29.99–1.99]; *p* = 0.08). In the SRT task, a significant difference was found regarding the time average reaction time (W = 49 [9.60–83.96]; *p* = 0.013), whereas it was not significant when considering the percentage of correct answers (W = 17 [− 0.99–4.00]; *p* = 0.22).

The ANCOVA conducted to address the perceptual and sustained alert components of the SRT task showed a significant difference between the PD patients and the healthy controls (F(1,15) = 15,613; *p* = 0.002; *η* ^2^ part =  0.565).

### Driving simulation

Results from statistical analyses of several driving performance metrics are presented in Table 4.

**Fig. 2:**
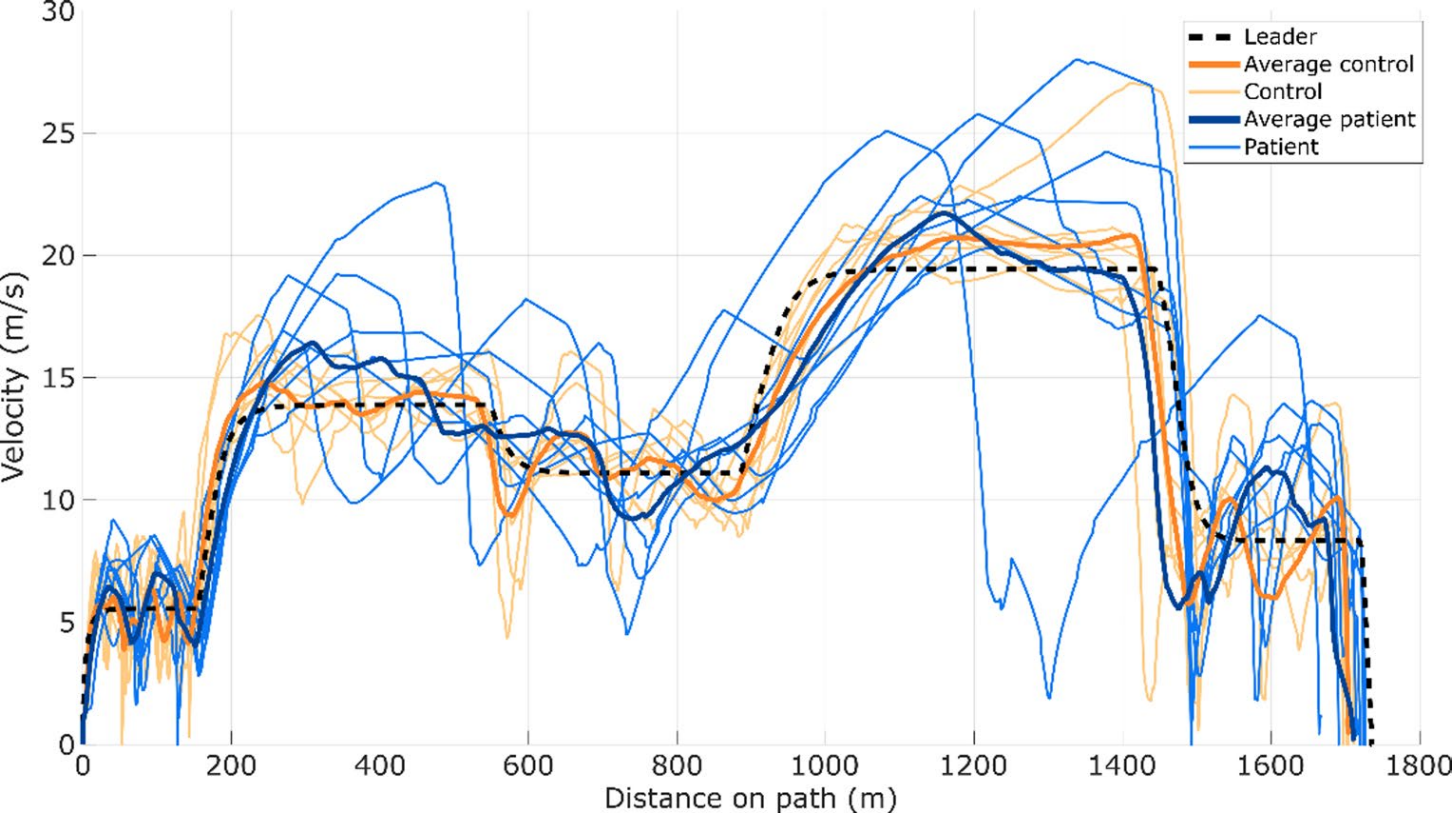
Velocity profiles as a function of distance in Experiment 2, comparing the leader (dashed black), control participants (orange), and patients (blue). Velocity profiles along the path in Experiment 2 for the leader (black-dashed), control group (orange), and patient group (blue). The leader profile shows the target trajectory and speed modulation, while the control group closely followed these patterns with relatively consistent velocities. In contrast, the patient group exhibited greater variability, with several deviations at different sections of the path. Higher dispersion was observed, especially in the segments between 400 and 800 m and 1200–1600 m, indicating less stable speed control compared to the control group.

Although statistical significance was not reached—likely due to the limited sample size and the proof-of-concept nature of this study—clear differences were observed between patients and controls in the reaction times of Experiment 1 and the driving speed in Experiment 2 (Fig. 2).

We found a relationship between the execution of the symbol search test and the performance in experiment 1 by a linear mixed model (estimate = − 0.2, *p* = 0.004*).

**Fig. 3:**
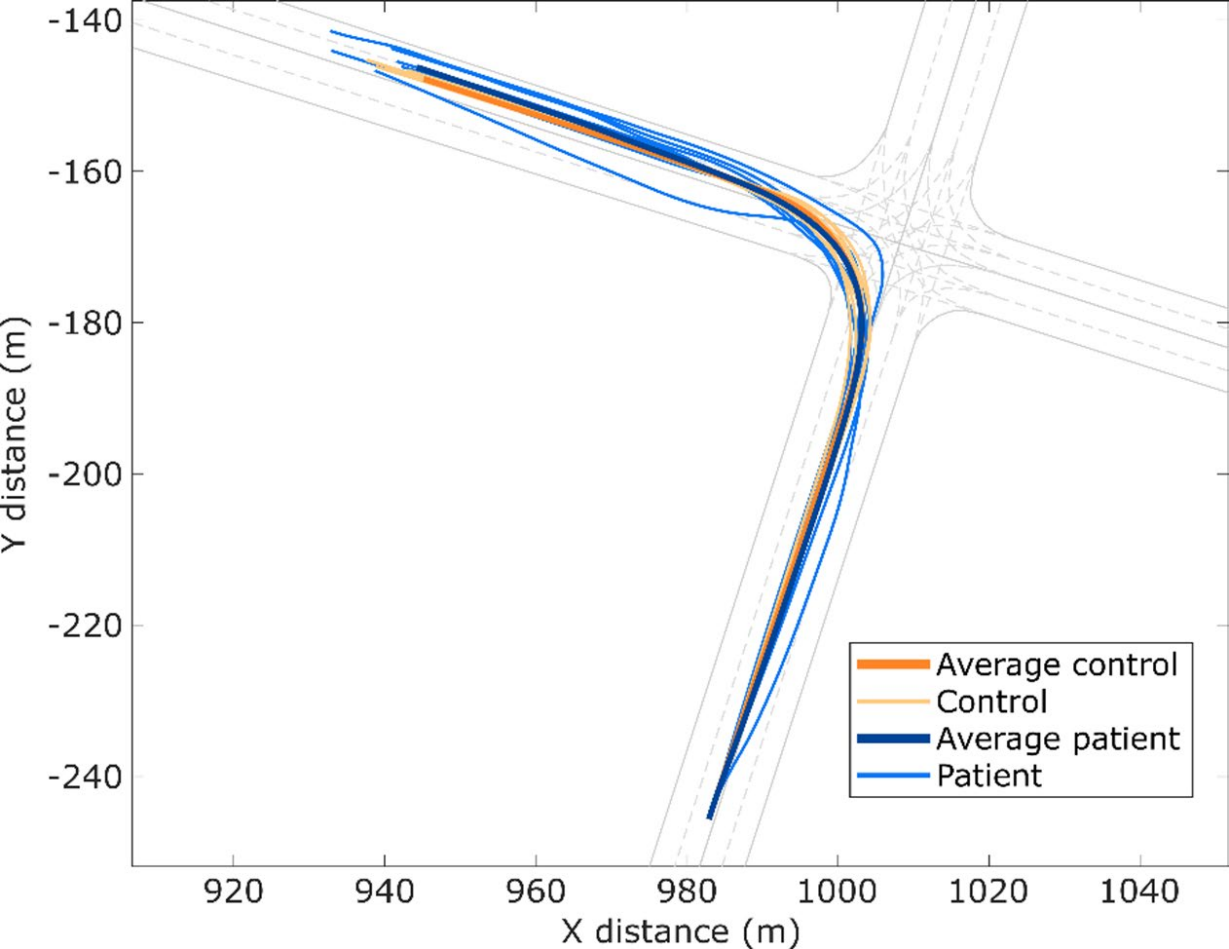
Paths in Experiment 4 for control (orange) and patient (blue) participants. Vehicle paths in Experiment 4 for the control group (orange) and patient group (blue) when negotiating a right-turn intersection. Both groups generally followed the intended turning trajectory, but patient trajectories displayed a wider spread, with some paths cutting closer to the inner curve or drifting toward the outer lane. This increased variability suggests reduced precision in lane-keeping during turning maneuvers among patients compared with control participants.

**Fig. 4:**
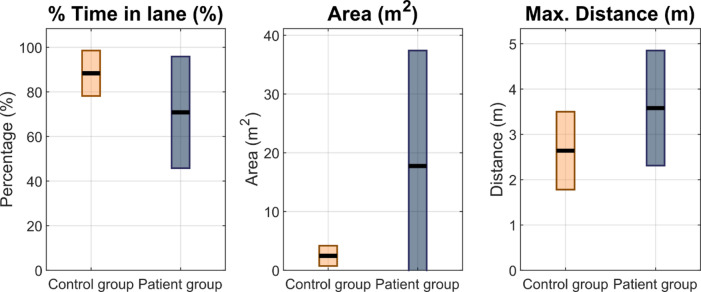
Box-plots comparison for experiment 4. Distributions of three performance measures are shown for the two experimental groups: control (orange) and patients(blue). Left panel: The time-in-lane percentage was higher and more consistent in the control group, whereas the patient group showed greater variability and lower median scores. Middle panel: The patient group exhibited a wider range and higher values in the total deviation area (area), suggesting less precise control. Right panel: Maximum deviation distance from the lane (max. distance) was greater in the patient group, indicating impaired steering accuracy. Each box plot displays the interquartile range, the mean (represented by the horizontal line), and whiskers indicating the whole range, excluding outliers.

Although differences in the ability to maintain lane position were observed in Experiment 3, they did not reach statistical significance. However, significant differences were found in Experiment 4 regarding both the maximum distance from the centre of the lane and area outside the lane (Figs. 3 and 4).

No significant statistical differences were found concerning experiment 5, although a difference between group means is noticeable.

**Table 4:** Differences between groups in driving simulation experiments.

Experiment	Metric	Control group	Patient group	*P* value	Statistic	Confidence interval
1	Reaction time (s)	2.02 ± 1.38	3.9 ± 2.36	0.113	1.621	[− 0.338, 3.074]
2	Reaction time (s)	4.26 ± 1.61	4.55 ± 1.53	0.657	0.450	[− 1.032, 1.611]
	Diff. velocity (km/h)	6.83 ± 2.63	9.81 ± 4.06	0.080	1.822	[− 0.382, 6.348]
	Distance (m)	56.34 ± 13.69	57.74 ± 30.94	0.906	0.119	[− 22.703, 25.507]
3	% Time in lane (%)	91.49 ± 15.17	83.47 ± 18.07	0.182	–1.358	[− 19.945, 3.914]
	Area (m^2^)	1.76 ± 3.21	6.23 ± 11.30	0.082	1.785	[− 0.590, 9.521]
	Max. distance (m)	2.24 ± 0.69	9.81 ± 4.06	0.386	0.877	[− 0.374, 0.947]
4	% Time in lane (%)	88.36 ± 10.19	70.84 ± 25.04	0.052	− 2.007	[− 35.163, 0.120]
	Area (m^2^)	2.46 ± 1.73	17.75 ± 19.65	0.013*	2.615	[3.471, 27.112]
	Max. distance (m)	2.64 ± 0.86	3.58 ± 1.27	0.039*	2.132	[0.050, 1.849]
5	% Time in lane (%)	95.01 ± 10.88	82.47 ± 30.21	0.242	− 1.188	[− 33.910, 8.809]
	Area (m^2^)	9.64 ± 30.47	79.90 ± 201.25	0.330	0.986	[− 73.808, 214.330]
	Max. distance (m)	2.62 ± 2.12	5.23 ± 8.86	0.411	0.830	[− 3.746, 8.971]

### Correlations

Significant correlations regarding PD evolution variables and PD treatment with simulation execution were found between reaction time in experiment 2 and levodopa dose (S = 102.42, *p* = 0.0211[− 0.97 to − 0.20], ρ=− 0.8288) and distance from the centre of the lane in experiment 4 with the levodopa dose (S = 12.611, *p* = 0.04077 [− 0.05 to 0.97], ρ = 0.77) and years of evolution (S = 6.56, *p* = 0.00845 [0.39–0.98], ρ = 0.88). Percentage of maintenance inside the lane in experiments 3 (S = 100.9, *p* = 0.0301 [− 0.97 to − 0.12], ρ=− 0.80) and 4 (S = 100.9, *p* = 0.0301 [− 0.97 to − 0.12], ρ = − 0.80) and area outside the lane in experiment 4 (S = 11.1, *p* = 0.0301 [0.12–0.97], ρ = 0.80) correlates with Hoehn & Yahr stage. This relationship indicates that disease progression is directly associated with a worsening of the ability to maintain vehicle trajectory and a centred lane position.

The cognitive components of the SRT task correlates with all the components considered in experiment 5: distance from the centre of the lane (S = 102, *p* = 0.0340 [− 0.98 to − 0.18], ρ = − 0.82), area outside the lane (S = 103.43, *p* = 0.0162 [− 0.98 to − 0.26], ρ = − 0.85) and percentage inside the lane (S = 8.57, *p* = 0.0162 [0.26–0.98], ρ = 0.85).

## Discussion

Parkinson’s disease affects multiple functional domains—including motor control, sensory integration, and cognitive processing, all of which are essential for maintaining safe driving behaviour.

PD patients are more prone to cease driving earlier than their contemporaries, as the disease progresses and associates a gradual decrease of their ability to drive^5,21^. According to a recent review^9^ the evaluation for driving fitness in PD patients should include 6 different aspects: general patient characteristics like age and Hoehn&Yahr stage, driving history, motor impairment, usually measured with the MDS-UPDRS-III; cognitive evaluation with neuropsychological tests and other PD-related symptoms like sleep disorders, motor fluctuations and adverse effects. All these data were included in our study in the initial evaluation of the participants.

When comparing the clinical interview and neuropsychological test results with the driving simulator data, we observed that standard cognitive assessments failed to detect significant differences between PD patients and controls, while the simulator revealed clear impairments in reaction time and visuospatial accuracy, both critical for safe driving. The inclusion of computerized tasks further supported these findings: participants with PD showed deficits in perceptual processing and sustained alertness during the SRT task, in line with previous studies^16,22–24^. These results suggest that traditional assessments may not capture subtle but functionally relevant cognitive alterations.

Notably, performance in Experiment 1, which was designed to assess sustained attention and processing speed, is predicted by performance on the Symbol Search test, as both tasks evaluate similar cognitive functions. Likewise, the association between SRT performance and reverse driving suggests that simple computerized reaction time tasks—relying on visual stimulus detection—may help predict functional abilities in real-world scenarios such as backing into a parking space, which also requires visual detection skills. These visuospatial impairments may also contribute to the increased difficulty observed when executing left turns, particularly among right-handed individuals, considering that the typical driving position in the vehicle is not centered but shifted to the left.

Our findings reinforce the growing evidence that standard psychomotor tests may be insufficient to detect the early-stage cognitive and visuospatial impairments that affect driving ability in PD^25^. Driving simulators, as used in this study, provide a multidimensional assessment environment that better reflects the real demands of driving.

The simulator used in this study was intentionally programmed to address domains known to be affected in early PD, such as sustained attention, visuospatial control, and reaction time. The scenarios were designed to simulate real-world driving tasks like left turns, lane positioning, and reverse driving, allowing for objective measurement of trajectory control and response time. These features were developed to reflect both clinical needs and the literature on common driving challenges in PD, and to go beyond the limited scope of standard cognitive assessments.

While driving simulators inevitably differ from real world driving due to the lack of full sensory feedback and risk perception, they offer a reproducible, safe, and ethically sound alternative for evaluating performance under controlled conditions. Moreover, their flexibility makes them valuable for future implementation of personalized driving assessments in clinical settings.

This study has several limitations. First, the sample size was relatively small, and participants were not randomly selected, which limits both the generalizability of the findings and the reliability of the statistical comparisons. As the main objective of this study was to assess the methodological feasibility and reliability of implementing a driving simulation protocol in individuals with Parkinson’s disease that still drive, rather than to perform hypothesis testing or draw generalizable conclusions, it can be achieved with this number of participants. Second, the sample was not fully balanced in terms of socioeconomic and educational background, which may influence neuropsychological performance. Third, although gender was matched across groups but the overall gender distribution may not reflect the general driving population. Even though participants were all male, this distribution may be explained according to the demographic characteristics of the population affected by Parkinson’s disease and of active drivers in Spain. According to data from the *Dirección General de Tráfico* (DGT, 2024), men aged 50 years and older hold driving licenses 46.6% more frequently than women of the same age group^26^. Furthermore, epidemiological data indicate that Parkinson’s disease is diagnosed approximately twice as often in men as in women^27^. Therefore, although the small and exclusively male sample limits the generalizability of the results, it remains representative of the population segment most likely to hold a valid driving license and to be affected by Parkinson’s disease. Finally, although simulators provide a valuable controlled environment, they cannot fully replicate the sensory, emotional, and contextual variables inherent in actual driving situations.

By bridging the gap between clinical evaluation and real-world functional performance, simulator-based assessments offer a crucial opportunity to redefine how we understand and measure driving fitness in neurodegenerative conditions like Parkinson’s disease.

## Conclusion

This proof-of-concept study highlights that routine cognitive assessments in patients with Parkinson’s disease may not always be efficient enough to detect impairments that could potentially compromise driving safety, such as visuospatial skills and sustained alert impairments. Previous studies have confirmed that this type of impairment can emerge in the early stages of the disease, even in the absence of other indicators of cognitive decline. While instrumental tests such as computerized reaction time assessments can detect these deficits, they are not currently used in real-life tasks like driving ability. The present study demonstrates that immersive driving environments targeting specific cognitive functions guided by the known characterized deficits of the disease may help identify deficits that go unnoticed with conventional assessments. Although the actual impact of these deficits on driving safety remains to be fully evaluated, the refinement of driving simulators—such as the one used in this experiment—represents a promising step toward improving the detection of variables that may affect driving performance in people with PD.

## Data Availability

The datasets generated and analyzed during the current study are available from the corresponding author on reasonable request.

## Declarations

### Reporting guidelines

This manuscript was prepared in accordance with the STROBE (Strengthening the Reporting of Observational Studies in Epidemiology) checklist for cross-sectional observational studies.
